# New science, drug regulation, and emergent public health issues: The work of FDA’s division of applied regulatory science

**DOI:** 10.3389/fmed.2022.1109541

**Published:** 2023-01-19

**Authors:** Kimberly Chiu, Rebecca Racz, Keith Burkhart, Jeffry Florian, Kevin Ford, M. Iveth Garcia, Robert M. Geiger, Kristina E. Howard, Paula L. Hyland, Omnia A. Ismaiel, Naomi L. Kruhlak, Zhihua Li, Murali K. Matta, Kristin W. Prentice, Aanchal Shah, Lidiya Stavitskaya, Donna A. Volpe, James L. Weaver, Wendy W. Wu, Rodney Rouse, David G. Strauss

**Affiliations:** ^1^Division of Applied Regulatory Science, Office of Clinical Pharmacology, Office of Translational Science, Center for Drug Evaluation and Research, United States Food and Drug Administration, Silver Spring, MD, United States; ^2^Booz Allen Hamilton, McLean, VA, United States

**Keywords:** regulatory science, drug regulation, public policy, public health, US Food and Drug Administration, FDA–Food and Drug Administration, translational science

## Abstract

The U.S. Food and Drug Administration (FDA) Division of Applied Regulatory Science (DARS) moves new science into the drug review process and addresses emergent regulatory and public health questions for the Agency. By forming interdisciplinary teams, DARS conducts mission-critical research to provide answers to scientific questions and solutions to regulatory challenges. Staffed by experts across the translational research spectrum, DARS forms synergies by pulling together scientists and experts from diverse backgrounds to collaborate in tackling some of the most complex challenges facing FDA. This includes (but is not limited to) assessing the systemic absorption of sunscreens, evaluating whether certain drugs can convert to carcinogens in people, studying drug interactions with opioids, optimizing opioid antagonist dosing in community settings, removing barriers to biosimilar and generic drug development, and advancing therapeutic development for rare diseases. FDA tasks DARS with wide ranging issues that encompass regulatory science; DARS, in turn, helps the Agency solve these challenges. The impact of DARS research is felt by patients, the pharmaceutical industry, and fellow regulators. This article reviews applied research projects and initiatives led by DARS and conducts a deeper dive into select examples illustrating the impactful work of the Division.

## Introduction

The United States Food and Drug Administration (FDA) recently highlighted regulatory science as an Agency priority in its “2021: Advancing Regulatory Science at FDA: Focus Areas of Regulatory Science (FARS)” report (1). Regulatory science is defined as the science of developing new tools, standards, and approaches to assess the safety, efficacy, quality, and performance of FDA-regulated products (2) and it plays an important role in supporting regulatory decision-making and policy development (3). Located within FDA’s Center for Drug Evaluation and Research (CDER), Office of Translational Sciences, Office of Clinical Pharmacology, the Division of Applied Regulatory Science (DARS) conducts mission-critical applied regulatory science research for the Agency.

Division of Applied Regulatory Science engages stakeholders in mission-critical laboratory, computational, and clinical applied research to improve regulatory decision-making and public health. DARS moves new science into the FDA review process and addresses emergent regulatory and public health questions. Capabilities within the Division include laboratory-based research specializing in omics, bioanalysis, microphysiological and cellular systems, immunology, and electrophysiology as well as *in silico* research performed by informatics and computational modeling groups. In addition, DARS conducts clinical research focused on facilitating new and biosimilar/generic drug development and assessing the safety of marketed drugs. Using applied research, DARS investigates questions related to clinical pharmacology, medical toxicology, systems pharmacology, chemistry, and biology (see Supplementary Appendix 1 for a more thorough description of DARS capabilities).

The research projects and initiatives led by DARS originate from a variety of sources. Some projects described in this article, such as those found under the Section “Emergent regulatory and public health questions,” were initiated following requests from FDA or CDER senior leadership in support of an Agency or Center priority. Other projects began as regulatory consult requests to DARS from other offices within the Agency.

Upon receiving a project request from senior leadership or a consult, the Division rapidly assembles a diverse, interdisciplinary team of experts. These scientific thought leaders have decades of cumulative experience across the translational research spectrum (4). Moreover, DARS scientists frequently collaborate on projects with leading academic, government, and private institutions (Supplementary Appendix 2).

An article (5) published in 2017 reviewed DARS’ mission and scope of work at that time, describing example research studies and projects. Since that time the Division has grown, as has the impact of DARS research on drug-related policies and regulatory decisions. This article reviews current DARS’ projects and initiatives, showcasing unique synergies and collaborations, and further examines selected projects that highlight DARS’ impact. In each of these examples, DARS led combinations of applied laboratory, computational and clinical research to provide key data to the Agency and to inform regulatory decisions or policy. The examples described in this article represent only a part of the Division’s full research portfolio.

## Emergent regulatory and public health questions

Two recent examples of DARS engagement on emergent regulatory and public health issues involved over-the-counter drug products that were widely available to patients (i.e., sunscreens and the heartburn medication ranitidine) and other examples involved the prescription drug, remdesivir, a treatment for COVID-19.

### Sunscreen active ingredients absorption

**FIGURE 1:**
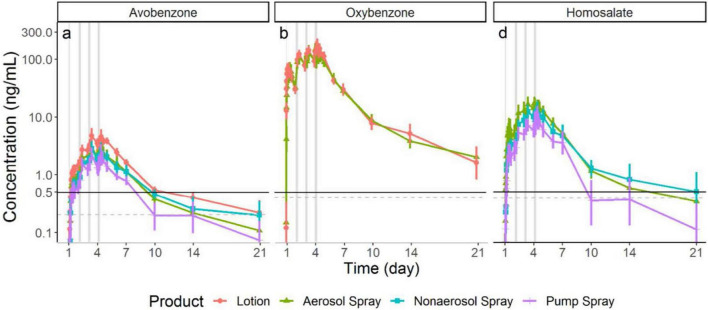
Division of applied regulatory science (DARS) conducted a clinical study to assess the systemic absorption and pharmacokinetics of sunscreen active ingredients. Study results (shown in these graphs) demonstrate these compounds are absorbed at levels that would trigger additional safety studies. Further research is needed and these findings do not indicate individuals should refrain from using sunscreen (7).

Food and Drug Administration issued a proposed rule (6) in February 2019 to update regulatory requirements for 16 sunscreen ingredients including recommendations to assess their human systemic absorption. Historical assumptions posited sunscreen active ingredients were not absorbed and that the clinical studies to test this were not feasible. In collaboration with FDA’s Office of New Drugs, DARS led and conducted clinical research demonstrating that maximum usage sunscreen clinical trials are feasible, that sunscreen active ingredients are absorbed at levels that would trigger additional safety studies (Figure 1), and that further research is needed to fill in data gaps for sunscreen ingredients. Results from these studies were published in the *Journal of the American Medical Association: JAMA* (7, 8), one of which was the most viewed *JAMA* article in 2019 (8). DARS also published the detailed bioanalytical method it developed (9) to enable further study and collaborated with FDA’s Office of Pharmaceutical Quality staff on a study to assess sunscreen ingredient absorption with an *in vitro* model (10).

### Over-the-counter heartburn medication ranitidine

**FIGURE 2:**
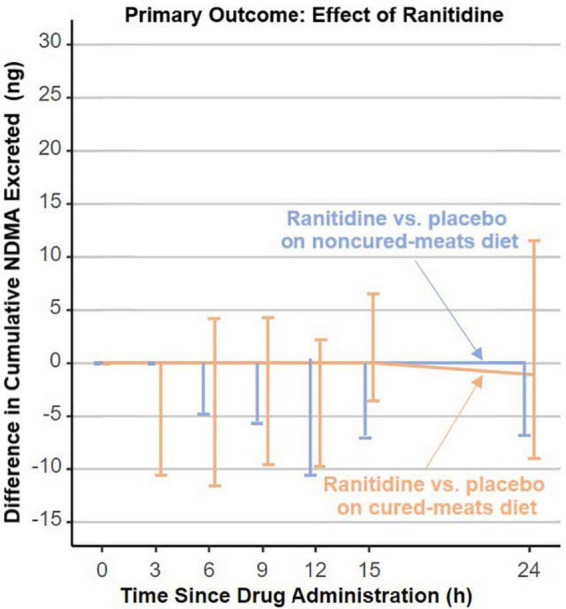
Division of applied regulatory science (DARS) designed a rigorous clinical study that carefully controlled for factors known to influence *N*-nitrosodimethylamine (NDMA) measurements including diet. The study found no evidence of increased NDMA content in participants’ urine who were administered ranitidine (12).

In 2019, FDA received a citizen petition (11) indicating that unacceptably high levels of *N*-nitrosodimethylamine (NDMA), a probable human carcinogen, were detected in specific ranitidine products. Ranitidine products were removed from the US market in April 2020, owing to the unacceptable amounts of NDMA in ranitidine products. Additionally, the petitioner postulated that ranitidine could convert to NDMA in people. In response, DARS, working with FDA’s Office of New Drugs and Office of Pharmaceutical Quality, designed a rigorous clinical study that carefully controlled for factors known to influence NDMA exposure and measurements including diet (12). DARS developed and validated a sensitive analytical method that was used to measure NDMA in study participants’ urine and blood. The study found no evidence of increased NDMA content in participants’ urine or blood (Figure 2) who were administered ranitidine (12). Additional *in vitro* investigations with simulated gastric fluid supported that ranitidine would not produce NDMA under physiologic conditions (13). With this information, FDA may consider allowing ranitidine products back on the market if they are manufactured to be stable with low, acceptable amounts of NDMA that do not increase over time.

### Adverse events of COVID-19 therapeutics

In October 2020, FDA issued an Emergency Use Authorization for remdesivir to treat COVID-19. Subsequent adverse event reports received by FDA suggested a possible connection to acute kidney injury or hepatic injury. FDA consulted DARS to assess whether there was additional evidence or a potential mechanism for these adverse events. DARS used target prediction software, secondary pharmacology data analysis, quantitative structure-activity models, and structural similarity analysis to review remdesivir and its metabolites. DARS found that remdesivir and its metabolites were structurally similar to drugs associated with renal and hepatic toxicity and a description of the potential risks associated with these adverse events is now found in remdesivir’s product labeling (14).

### Optimizing COVID-19 therapies

Division of Applied Regulatory Science developed and published a mechanistic COVID-19 disease model that can be used to guide candidate drug selection and dosing strategies based on non-clinical data. The model strategy was developed using remdesivir as a proof-of-concept example and provides a well-calibrated and validated model for performing similar non-clinical to clinical translations for other potential COVID-19 therapies (15).

## Addressing the opioid epidemic

Prescription opioids are powerful pain-reducing medications that have both benefits and potentially serious risks (16). The U.S. is in the midst of an opioid epidemic. Nearly 92,000 people died from drug-involved overdose in 2020, 75% of which involved an opioid (17). FDA leaders committed the Agency to work proactively toward a solution (18) and enacted the 2016 Opioid Action Plan (1) outlining concrete steps the Agency would take to address the epidemic. In support of the Agency’s efforts, DARS uses its diverse expertise to help fight this public health crisis.

### Optimizing opioid reversal agents

**FIGURE 3:**
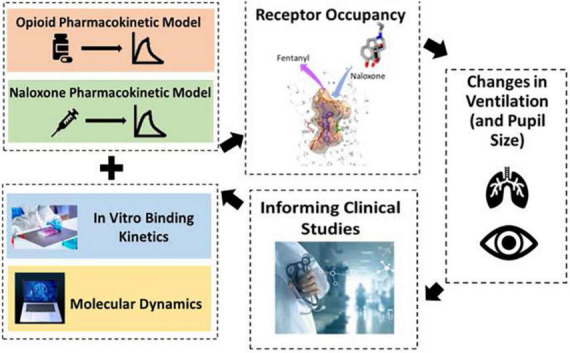
To help combat the opioid epidemic, division of applied regulatory science (DARS) is conducting research to optimize the use of opioid antagonists and to advance drug development tools to support new opioid antagonists. DARS is using a variety of methods including molecular dynamics, computational modeling, and clinical studies.

Division of Applied Regulatory Science is conducting *in vitro*, computational, and clinical research to optimize the use of existing opioid antagonists (naloxone) and to advance drug development tools for new opioid antagonists (Figure 3). Synthetic opioids, such as illicitly manufactured fentanyl, cause a large number of opioid overdose deaths in the U.S. (19). Moreover, a rise in illicit fentanyl derivatives (that vary widely in potency) make it difficult to identify an adequate naloxone dose. DARS, in collaboration with the University of Maryland (20, 21), applied an advanced *in silico* molecular dynamics method called metadynamics to elucidate the dissociation mechanism of fentanyl and its derivatives to calculate the residence times at the mu-opioid receptor. The simulations uncovered two distinct dissociation mechanisms, one of which involves a newly identified binding pocket that contributes to the long residence time and high binding affinity of fentanyl and its derivatives. This new method was used to predict the relative dissociation time of a newly identified opioid that emerged from illegal markets, helping to inform overdose prevention strategy (22).

Division of Applied Regulatory Science developed a quantitative systems pharmacology computational model (23) to assess naloxone dosing strategies and applied it to the regulatory review of a new naloxone formulation for use by military personnel and chemical incident responders (24, 25). The model connects the pharmacokinetics of different opioids and naloxone to *in vitro* receptor binding kinetics, and subsequently to effects on respiratory depression and cardiac arrest. DARS used this model to assess the impact of naloxone dosing administered as an emergency treatment or temporary prophylaxis following exposure to fentanyl or carfentanil using endpoints of respiratory depression and cardiac arrest (24, 25). With this information, FDA’s review team was better able to understand this naloxone formulation’s capacity to reverse opioid effects under different scenarios.

Division of Applied Regulatory Science conducted a clinical study to characterize intranasal naloxone exposure after different repeat dosing strategies and predict the impact of the different dosing strategies on rescuing patients from fentanyl and fentanyl-derivative overdoses using the computational model described above (26). In addition, through a collaboration with Leiden University, an additional study is assessing the dynamics of how intranasal naloxone reverse respiratory depression from fentanyl in healthy opioid naïve participants and chronic opioid users (27). Together, these studies will inform intranasal naloxone dosing strategies in the community setting.

### Opioid drug-drug interaction effects on respiration

**FIGURE 4:**
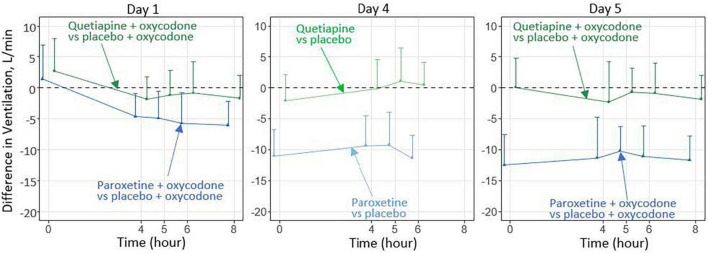
In this preliminary study involving healthy participants over 5 days, paroxetine combined with oxycodone, compared with oxycodone alone, significantly decreased the ventilatory response to hypercapnia (30), whereas quetiapine combined with oxycodone did not cause such an effect. Additional investigation is needed to characterize the effects after longer-term treatment and to determine the clinical relevance of these findings.

In 2016, FDA issued a warning about the increased risk of respiratory depression when combining opioids with benzodiazepines (28). Following the warning, DARS received a consult request concerning the potential for drug-drug interactions between opioids and drugs that might be co-prescribed *in lieu* of benzodiazepines. DARS conducted non-clinical *in vivo* studies to assess effects on respiratory depression for drugs given alone or in combination with an opioid (29). As a result of these studies, DARS designed a clinical study to assess the translatability of the findings to humans (27). This DARS-led clinical study assessed whether combining the selective serotonin reuptake inhibitor paroxetine or the atypical antipsychotic quetiapine with the opioid oxycodone, compared to oxycodone alone, decreased ventilation during hypercapnia (elevated carbon dioxide). The study found that paroxetine combined with oxycodone decreased ventilation (Figure 4), indicating the need for further study of the clinical implications (30).

### Public Health Assessment *via* Structural Evaluation (PHASE)

**FIGURE 5:**
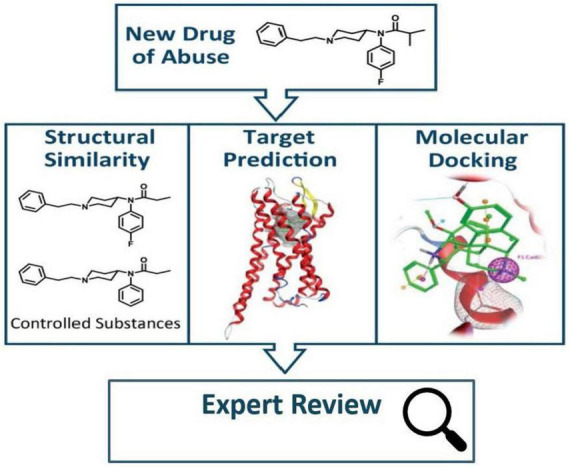
When a new drug of abuse is identified, public health assessment *via* structural evaluation (PHASE) uses chemical structure to assess the new drug’s risk to public safety (31).

Division of Applied Regulatory Science developed a Public Health Assessment *via* Structural Evaluation (PHASE) methodology that uses molecular structure to predict the biological function (31) of newly identified opioids (Figure 5). Results from PHASE have the potential to inform public health and law enforcement agencies with vital information regarding newly emerging illicit opioids in the absence of pharmacological data. In an example of using this method, DARS assessed kratom alkaloids (32), which was highlighted by then FDA Commissioner Dr. Scott Gottlieb in a statement about kratom’s potential for abuse, addiction, and serious health consequences (33).

## Better predicting potential drug safety issues

In 2015, FDA published “Assessing CDER’s Drug Safety-Related Regulatory Science Needs and Identifying Priorities” (34, 35). One of the priorities was to develop and improve predictive models of safety in humans (34). DARS is pursuing multiple projects to assess new tools to help identify, predict, assess, and manage drug-related adverse events that can be incorporated into the drug safety evaluation process.

### Predicting immune-mediated adverse events

While significant progress has been made in engineering biological products, the human immune system may still produce an unexpected or exaggerated immune response to a drug product resulting in poor efficacy or life-threatening adverse reactions. DARS is comparing the ability of different non-clinical models to predict immune-mediated adverse events from biological drug products. DARS tested the use of novel non-clinical models to predict cytokine release syndrome, a potentially life-threatening complication associated with biological products (36, 37) and showed that non-clinical models can effectively demonstrate this adverse event. Additionally, after successfully demonstrating immune-mediated activation in a non-clinical model (38), investigations are continuing for checkpoint inhibitor oncology treatments for which adverse events cannot presently be predicted using computational, *in vitro*, or conventional non-clinical methods.

### Leveraging molecular target information to predict safety issues

Knowledge of a drug’s molecular targets can provide early identification of a drug’s effects and potential safety concerns. DARS leads multiple efforts collating information about a drug’s known and predicted targets to identify potential safety concerns. For example, DARS developed multiple computational methodologies, including with machine learning, to predict a drug’s adverse effects based on the biological receptors that the drug, or similarly structured drugs, are known to target (39–41). These computational methodologies demonstrated promising performance in predicting significant adverse events. These results may indicate which organ systems and adverse event categories to closely monitor during clinical trials or during clinical and non-clinical data review.

**FIGURE 6:**
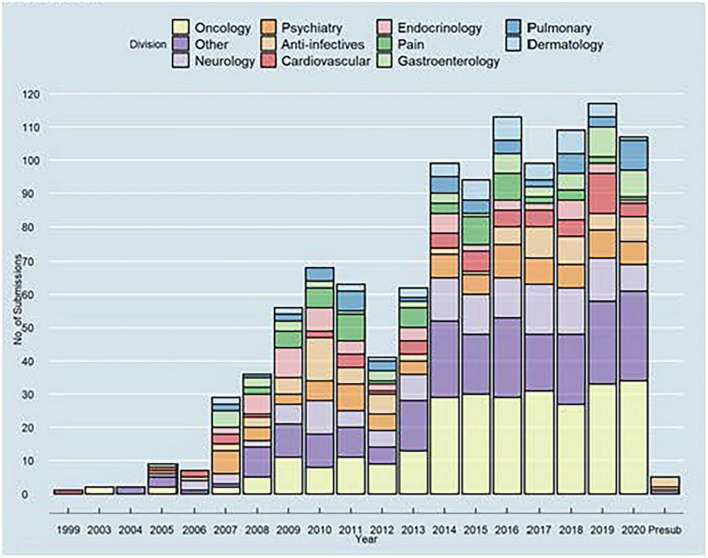
Division of applied regulatory science (DARS) manually extracted data from a variety of secondary pharmacology reports submitted by sponsors. The extracted data was then curated into a database (43) to allow for easier access and analysis by the agency.

Additionally, DARS is analyzing and building a database (Figure 6) for secondary pharmacology activity submitted by industry as part of their Investigational New Drug application (42, 43). A drug developer typically conducts *in vitro* target binding and functional assays for 80–100 biological receptors to determine potential on-target and off-target effects. However, the targets chosen for the assays as well as the submission format are not currently standardized across the industry. Data from these assays have been manually extracted and curated into a database that will allow easier access to and analysis of these study results. Additionally, DARS is engaging in a public-private partnership with the Pistoia Alliance to determine the best methods for future regulatory submission of these studies.

### Predicting drug interactions with (quantitative) structure activity relationship [(Q)SAR] models

The use of (quantitative) structure-activity relationship, or (Q)SAR, models has become an integral part of regulatory review as it can rapidly assess a compound’s toxicological and pharmacological properties based solely on chemical structure (5, 44). (Q)SAR models describe the association between chemical structural features and biological activity under the general assumption that similar chemical structures exhibit similar biological activities (45). (Q)SAR models and databases developed through DARS are used to support drug safety assessments and inform regulatory decisions at FDA. Models for several endpoints that comprise the standard genetic toxicity battery described in the International Council for Harmonisation (ICH) S2(R1) regulatory guidance (46) were recently updated and best practices on their use were also described (47–49). Additionally, a structure-activity relationship (SAR) profiler was constructed through an external collaboration to evaluate potential interactions caused by metabolites. Lastly, DARS recently developed a new model to predict a drug’s potential to cross the blood-brain barrier (50) and is in the process of developing a model to predict drug-induced cardiotoxicity using post-market safety data.

### Drug-drug interaction studies

As patients often use more than one drug at a time, it is critical to know if drugs taken together interact leading to safety or efficacy implications. To collect this information, FDA requires drug-drug interaction studies from pharmaceutical sponsors (51). DARS evaluates *in vitro* and *in vivo* methods to identify best practices when conducting transporter- or metabolism-based drug-drug interaction studies. For example, conflicting information appears in the scientific literature regarding the extent of specific cytochrome P450 (CYP) involvement in metabolizing methadone (a treatment for opiate dependence). Drawing from previously submitted new drug applications, DARS constructed a database of drug-drug interaction studies between methadone and antiviral medications known to affect these CYP enzymes (52, 53). After analyzing these 29 studies, DARS formulated recommendations on how best to conduct future drug-drug interaction studies with methadone.

## Facilitating biosimilar and complex generic development

Bringing more drug competition to the market and addressing the high cost of medicines is a priority for FDA and the Department of Health and Human Services (54). Improving the efficiency of biosimilar and generic drug development can facilitate robust drug market competition and help reduce drug costs. Demonstrating the Agency’s commitment, FDA announced the Drug Competition Action Plan (54) in 2017 and the Biosimilars Action Plan (55) in 2018. The plans outlined concrete steps the Agency would take to remove barriers to biosimilars and generic drug development. This included prioritizing biosimilars and complex generic drug applied research projects.

### Pharmacodynamic biomarkers for biosimilar development and approval

**FIGURE 7:**
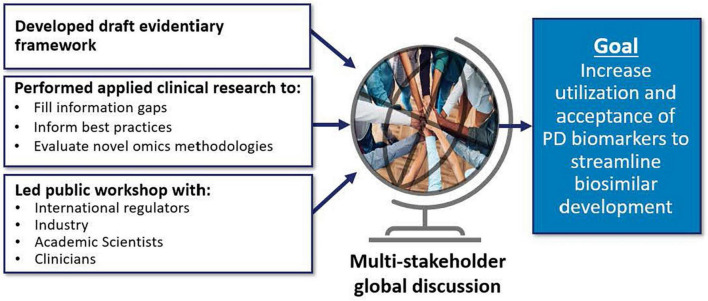
Many of division of applied regulatory science (DARS’) current initiatives will inform the agency’s thinking on the use of pharmacodynamic (PD) biomarkers to demonstrate biosimilarity that may streamline or negate the need for comparative clinical studies (56, 57). This includes conducting applied clinical research (58–60) to fill information gaps, inform best practices, and evaluate novel omics methodologies (61). DARS also developed an evidentiary framework, the draft version of which was presented at a DARS-led public workshop (62).

Many of DARS’ current initiatives (Figure 7) will inform the Agency’s thinking on the use of pharmacodynamic (PD) biomarkers to demonstrate biosimilarity that may streamline or negate the need for comparative clinical studies (56, 57). This included conducting three clinical studies (58–60) to define best practices on characterizing PD biomarkers for different classes of drugs and to develop general considerations applicable to all types of biomarkers for biological products. These studies included assessments to evaluate uses of proteomic and transcriptomic analysis of human plasma to identify novel biomarkers for biosimilar development (61). A joint FDA/Duke Margolis Workshop (62) discussed initial findings and facilitated a broader discussion on use of PD biomarkers for biosimilar development. Additional details will be reported in the January 2023 themed issue on Innovations in Biosimilars in the journal *Clinical Pharmacology and Therapeutics* (61, 63).

### Novel methods to assess immunogenicity for biosimilars and complex generic drugs

A factor influencing rapid development of biosimilars and generics is the ability to predict the potential risk of a stronger immune response (immunogenicity) in humans to a proposed biosimilar or generic drug than that seen with the innovator product. For most biosimilars this is still assessed through clinical trials. DARS is studying the ability of non-clinical approaches to predict immunogenicity risk without conducting a clinical trial. This includes assessing specific *in vitro* assays and cell types, *in vivo* models, and identification of useful controls. These methods have the potential to identify products with immunogenicity risk sooner and streamline the development of biosimilars and certain complex generic drugs.

### Supporting generic drug development in pediatrics

**FIGURE 8:**
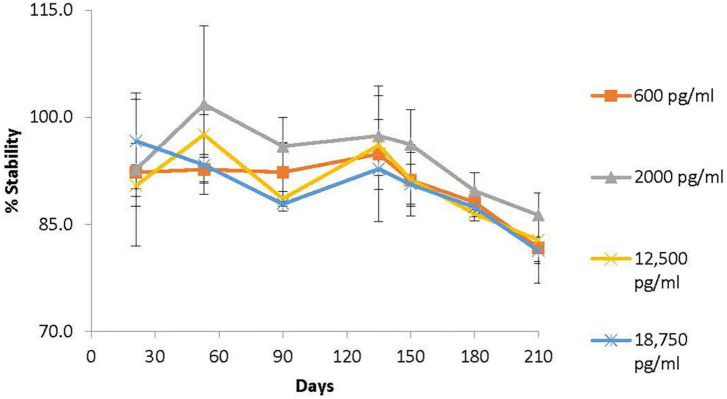
Division of applied regulatory science (DARS) developed and validated novel, sensitive, and specific analytical methods to support pediatric pharmacokinetic studies using dried blood spot (DBS) cards. One such method was for methylphenidate, an attention-deficit hyperactivity disorder drug. The long-term stability profile of methylphenidate on DBS cards stored for up to 7 months at room temperature (64) is shown. Methylphenidate was found to be stable (±15% of spiked concentration) on DBS cards stored at 24°C for up to 5 months.

Performing clinical studies in pediatric populations that involve frequent blood samples can be challenging if conventional methods are used that require relatively large blood volumes that can limit generic drug development for children. An alternative method is determining the concentration of drug in blood from dried blood spots (DBS) that only require 10 μl of blood compared to ∼5 mL (500 times more) with conventional methods. However, there has been a lack of analytical methodology metrics for using DBS analysis for generic drug bioequivalence studies. DARS developed and validated novel, sensitive, and specific analytical methods (Figure 8) to support pediatric pharmacokinetic studies using DBS cards (64).

### Investigating novel approaches to establish bioequivalence for complex generic products

In collaboration with CDER’s Office of Generic Drugs, DARS has conducted multiple studies to evaluate the potential of new approaches to advance the development of complex generic drugs such as injectable delayed release formulations and device-drug combinations. DARS conducted a non-clinical *in vivo* study to evaluate an *in silico* modeling approach for delayed-release risperidone injectable products that might be used to inform clinical trials for generic drug approvals. Another recent study investigated slow-release dexamethasone intravitreal implants used to treat inflammatory conditions in a wide variety of ocular diseases. Unfortunately, no generic implants are available on the market largely due to the challenges associated with measuring drug concentration within the eye to establish bioequivalence. DARS conducted a non-clinical *in vivo* study to measure and correlate intraocular and systemic dexamethasone concentrations after intravitreal implantation (65). This research will inform whether systemic dexamethasone exposure might serve as a surrogate for intra-ocular exposure in assessing the bioequivalence of generic dexamethasone implants. A similar approach may be possible for development of other generic intraocular products.

## Advancing therapies for rare diseases

**FIGURE 9:**
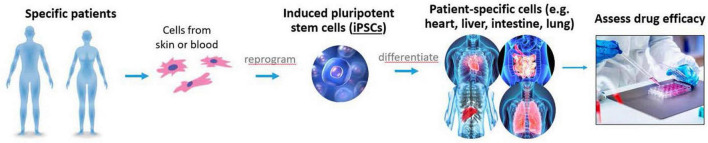
Complex *in vitro* models, induced pluripotent stem cells (iPSCs), and other non-clinical methods are some of the tools being evaluated by division of applied regulatory science (DARS) to help advance therapies for rare diseases.

Food and Drug Administration is committed to advancing the development of therapies for rare diseases (66). Although a single rare disease is defined as affecting less than 200,000 people, collectively, over 7,000 rare diseases affect more than 30 million patients in the US (67, 68). With small individual patient populations, developing therapies for rare diseases faces unique challenges such as inadequately powered trials and restricted clinical study designs. FDA recognizes the need for new and novel drug development tools (Figure 9) as well as regulatory flexibility for the evidence required for drug approval (69).

### Drug approvals using non-clinical data

Division of Applied Regulatory Science led regulatory reviews of *in vitro* data to expand the approval of rare disease treatments. The scientific framework enables the evaluation of drug approval proposals in disease population subsets with very rare genetic variants who may not be well represented in clinical trials (70, 71). In 2017, DARS was consulted to determine if electrophysiology and other *in vitro* data were adequate to support the expanded approval of ivacaftor to treat patients with cystic fibrosis (CF) who have such rare variants that the patient population is too small for an adequately powered clinical trial. An *in vitro* cell-based approach was used to assess the response of the mutated or dysfunctional proteins in the presence of drug to make inferences about the potential for response in patients. As a result, the 2017 expanded approval of ivacaftor has allowed ∼1,500 new patients access to the drug based on *in vitro* data that predicted the clinical responsiveness of patients not included in clinical trials (72). Soon after, DARS evaluated *in vitro* data to support the expansion of approval for the combination drug ivacaftor/tezacaftor/elexacaftor (73). In 2018 DARS was also consulted to determine if *in vitro* functional data was adequate to support the approval of migalastat to treat patients with Fabry disease. An estimation about the extent of patient access to migalastat based on the original approval is not yet available but a similar increase in access to the drug for patients with 348 amenable *galactosidase alpha gene (GLA)* variants is expected (71). In 2021, *in vitro* data was also evaluated by DARS to support addition of two new amenable GLA variants to the label.

### Assessing the potential for iPSCs to streamline drug development for rare diseases

Induced pluripotent stem cells (iPSCs) may serve as a renewable source of differentiated cell types with patient- or population-specific properties (74–76). This model could replace, reduce, or help refine clinical trials for rare diseases. A collaboration with Stanford University is evaluating and comparing *in vitro* models with patient-specific iPSCs from patients with Duchenne Muscular Dystrophy and iPSCs genetically engineered to have the same genetic variants using CRISPR. This project seeks to inform the development of general standards, quality control criteria, and best practices for iPSC-based models to assess the efficacy for rare disease treatments.

### Identifying molecular targets for pediatric cancer

Pediatric cancers are rare diseases with limited treatment options (77). To facilitate drug development in accordance with the Research to Accelerate Cures and Equity (RACE) Act, FDA developed the Pediatric Molecular Target List to provide guidance to pharmaceutical developers when planning new drug and biologic submissions that may be relevant to pediatric cancer treatment. To assist with maintaining the Pediatric Molecular Target List, DARS is developing natural language processing algorithms to identify evidence in peer-reviewed literature and external databases for molecular targets associated with pediatric cancer. These algorithms will inform regular updates to the Pediatric Molecular Target List including identifying new references for existing targets, emerging evidence for new targets, as well as supporting the review of initial pediatric study plan submissions.

## New alternative methods

### New alternative methods to animal testing

**FIGURE 10:**
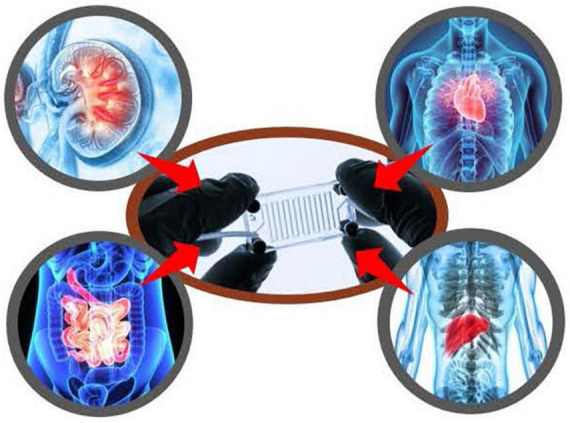
Division of applied regulatory science (DARS) is studying the utility of complex *in vitro* models, including with induced pluripotent stem cells (iPSCs) and microphysiological systems to reduce and replace animal testing.

As a part of FDA’s mission to ensure the safety and efficacy of human drugs, FDA reviews drug developer submitted data to establish under what conditions a new drug can be safely administered to patients and whether the new drug carries an increased risk of various adverse effects. This involves assessing end points that cannot be ethically obtained in humans, such as histopathological analysis of all major organs. Animal studies have played a critical role to meet this need and bring safe and effective therapies to patients. At the same time, FDA has a long-standing commitment to replace, reduce, and refine animal testing (the “3Rs”) with successes to date in harmonizing regulatory guidelines with international regulators and accepting alternatives to animal testing in certain areas (78, 79). Recent advances in systems biology, iPSCs, engineered tissues and mathematical modeling present new opportunities to improve our ability to predict risk and efficacy (Figure 10). However, multiple steps are required to translate these new technologies into regulatory use and maintain the same standard of safety, efficacy, and quality of FDA-regulated products. While we are nowhere near being able to replace all animal testing, there are opportunities for new alternative methods to make additional inroads in addressing the 3Rs for specific contexts of use. FDA has proposed funding for an Agency-wide New Alternative Methods Program, which was presented to the FDA Science Board in June 2022 by DARS (80).

In line with the Agency’s broader goals, DARS is performing applied research on complex *in vitro* models, including with iPSCs and microphysiological systems, to fill information gaps about the potential utility of these assays for regulatory contexts of use and to assess reproducibility, quality control and performance criteria. This has included studies on liver microphysiological systems (81, 82) for assaying drug toxicity, metabolism and accumulation, and additional studies with iPSC-derived liver and cardiac cells (74–76). Furthermore, DARS is assessing drug permeability and metabolism using the lung, gut, and a gut-liver interconnected MPS. Recently, DARS contributed to two white papers, which focused on *in vitro* methods for assessing drug-induced effects on cardiac contractility (83, 84). Additionally, DARS is studying the reproducibility of three-dimensional engineered heart tissue models. This work will be used to inform policy and guidance around qualifying alternative methods for regulatory use.

### Implementing regulatory science: From applied research to leading international regulatory guideline updates

**FIGURE 11:**
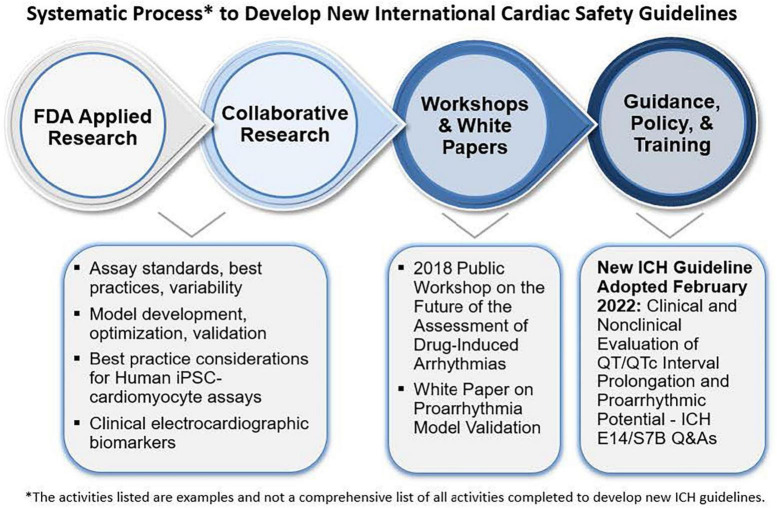
Under the comprehensive *in vitro* proarrhythmia assay (CiPA) initiative, division of applied regulatory science (DARS) (along with colleagues from CDER’s office of new drugs) developed a non-clinical model (94) to evaluate the risk of drugs causing abnormal heart rhythms with a high level of predictivity. DARS also leads research in collaboration with external consortia to overhaul the approach to assessing the risk of abnormal heart rhythms for all new drugs and update regulatory guidelines.

Division of Applied Regulatory Science has pursued a multi-year collaborative effort to improve the assessment of drug-induced cardiac toxicity from abnormal heart rhythms. Multiple drugs were removed from the market in the 1990s to 2000s and the ICH of Technical Requirements for Pharmaceuticals for Human Use Guidelines were implemented in 2005 to require specific non-clinical *in vitro* and *in vivo* studies, as well as dedicated clinical trials to assess the risk. However, these methods lacked specificity, resulting in drugs being dropped from development, sometimes unnecessarily. In response, the Comprehensive *in vitro* Proarrhythmia Assay (CiPA) initiative was implemented by FDA in collaboration with public-private partnerships including industry, academics, and other global regulators (Figure 11) (85–87). Through this, DARS led collaborative studies to assess:

• *in vitro* ion channel assay standards, best practices, and variability (88–90),
• *in silico* computational model development, optimization, and validation (91–96).
• best practice considerations for human iPSC-cardiomy-ocyte assays (97–99), and
• clinical electrocardiographic biomarkers (100–107).

The initiative prompted the ICH to open an Implementation Working Group with the purpose of developing a new guideline through a series of questions and answers to the existing ICH cardiac safety guidelines for an integrated strategy on using non-clinical data to inform clinical decision making. DARS staff served as lead (rapporteur) in developing new Questions and Answers (Q and As) to the ICH E14/S7B “Clinical and Non-clinical Evaluation of QT/QTc Interval Prolongation and Proarrhythmic Potential” guideline (108). This new ICH guideline contains best practice recommendations for *in vitro* ion channel and human induced pluripotent stem cell assays to enable use as follow-up studies in place of potential animal studies and principles for validating *in vitro* and *in silico* proarrhythmia models and qualifying them for regulatory use, which can reduce animal use.

## Limiting drug impurities and extractable/leachable compounds

**FIGURE 12:**
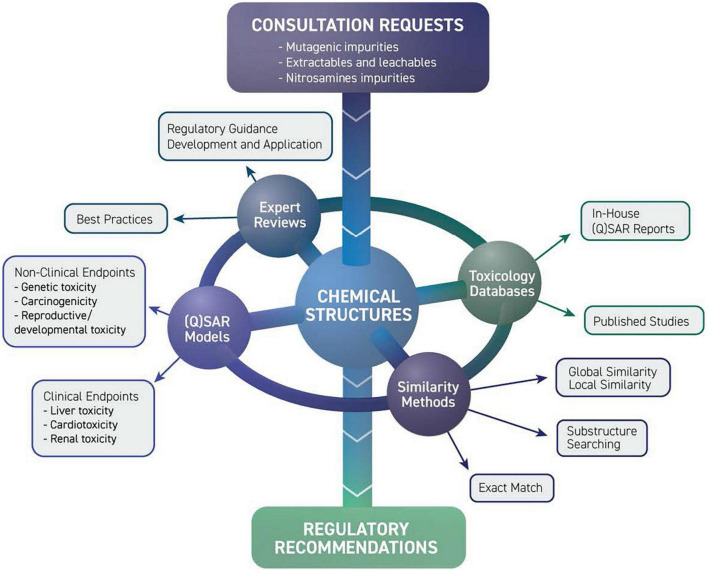
Limiting drug impurities and extractable/leachable compounds division of applied regulatory science (DARS) computational toxicology consultation service receives consult requests from across CDER. Experts review model predictions using a combination of toxicology databases, similarity methods, and (quantitative) structure-activity relationship [(Q)SAR] models before a regulatory recommendation is made.

The growing use of (Q)SAR models for the safety assessment of drug products led to the formation of the DARS Computational Toxicology Consultation Service (109). DARS staff receive consult requests from across CDER to support hundreds of new and generic drug applications, post-market safety assessments, and drug monograph reviews each year (Figure 12). For substances with limited experimental toxicology data, DARS reviews (Q)SAR model data submitted by the pharmaceutical applicant and, if needed, generates (Q)SAR predictions to inform setting an acceptable limit for impurities and extractable or leachable substances. Model predictions are then reviewed by DARS staff with the application of expert knowledge (110, 111) before a regulatory recommendation is made (109). Three common review scenarios for (Q)SAR consultation requests are described below.

### (Q)SAR assessments for drug impurities

The ICH M7(R1) international regulatory guideline recommends the use of (Q)SAR predictions as a state-of-the-art alternative to experimental testing to qualify a drug impurity for mutagenic potential (112). DARS receives consults to review mutagenicity (Q)SAR predictions in pharmaceutical industry submissions and to generate in-house (Q)SAR predictions using a battery of complementary models and expert knowledge.

### Structure-activity relationship assessments for extractable/leachable compounds

Extractable/leachable compounds are industrial chemicals that may be present in a drug product from manufacturing, packaging, drug delivery, and/or container closure systems. For non-mutagenic compounds with limited general toxicity data, a manual structure-activity relationship, or “read-across,” assessment based on structurally similar surrogate compounds may be used to inform setting a permissible daily exposure limit. DARS staff review applicants’ proposed surrogates for extractable/leachable compounds and, if needed, recommend alternative surrogates based on structural, metabolic, and physicochemical considerations.

### (Q)SAR evaluation of nitrosamine impurities to assess carcinogenic potency

Nitrosamine impurities constitute a special class of potentially mutagenic impurities that are controlled to very low levels because of their high carcinogenic potency. Due to the limited availability of experimental data for these impurities, read-across and (Q)SAR models may be used to inform an acceptable intake based on structurally related surrogate compounds. DARS staff conduct read-across and (Q)SAR analyses and review structure-based justifications for proposed nitrosamine limits to support internal decision-making.

## Additional regulatory challenges

The research topics and projects selected for this article represent only a part of DARS’ research portfolio and the Division’s work covers the full spectrum of regulatory science. To help further illustrate the breadth and depth of DARS’ work, this section highlights an additional sampling of projects.

### Drug overdose labeling

Within drug labeling, the overdosage section describes the signs, symptoms, and laboratory abnormalities that occur with drug overdose as well as recommendations on treatment, if known (113). Although FDA has implemented initiatives to update this section, it is typically not revised after initial approval (114). Using natural language processing informatics tools, DARS is identifying outdated or inaccurate information within the overdosage section. Thus far, the project has evaluated drugs from 10 different drug classes highly associated with drug overdose fatalities to inform potential label updates.

### New model to measure emergence of antimicrobial resistance

Antimicrobial resistance (AMR) remains a significant global public health threat. FDA, in collaboration with internal stakeholders, develops approaches to detect, prevent, and limit the impact of AMR (115). In particular, DARS developed a cell-based method to measure the rate at which antibiotic resistance appears. This hollow fiber bioreactor system (116) administers drugs to cells using human pharmacokinetic profiles to identify drug combinations that slow the development of resistance. Next-generation sequencing is being used to investigate the genetic and epigenetic biomarkers of antibiotic resistance and to investigate the effects of combinations of oral antibiotics on bacterial genomes and the gut microbiome (117) high-through put screening assay to identify optimal drug combinations for different organisms.

## Resulting regulatory impact

As mentioned previously, DARS comprises scientific thought leaders with decades of cumulative experience across the translational research spectrum. With their expertise, DARS staff not only lead cutting-edge research projects, but also lead or participate in multiple working groups and task forces. These in turn lead to the development of or significant changes to regulatory policies, decisions, and regulatory guidance. Below are examples.

### Supporting regulatory action: Drug labeling change

Division of Applied Regulatory Science generates data and evidence to help the Agency make the most well-informed regulatory decisions and take the most appropriate regulatory actions. Examples include issuing Drug Safety Communications, drug approvals, and as in this case, updating drug labeling. In 2018, information emerged showing a disproportionate number of neural tube defects in infants born in an HIV patient population treated with dolutegravir as part of a clinical trial (118). Over time, DARS received four consults on this issue. DARS evaluated dolutegravir in comparison with four related drugs using structural similarity, secondary pharmacology, and computational toxicology. The work informed updates to dolutegravir’s label and the labels of related drugs.

## Affecting regulatory policy: Drug development tools

Division of Applied Regulatory Science helps establish and steer policies surrounding drug development tools. DARS leadership serves on the agency’s Drug Development Tools Committee, the decisional body for qualification submissions. Many of DARS staff serve as expert reviewers for drug development tools qualification submissions. Additionally, DARS work is informing best practices and quality control criteria for complex *in vitro* models in support of biomarker qualification and the new Innovative Science and Technology Approaches for New Drugs (ISTAND) pilot program. DARS is also leading the development of an evidentiary framework to advance the use of PD biomarkers for biosimilars. The draft version of this evidentiary framework was presented at a DARS-led public workshop (119) and a review describing this work was recently published (63).

## Conclusion

These selected research topics and projects were chosen to represent DARS’ portfolio. The Division’s work in regulatory science plays an integral part in helping the Agency continually modernize its policies and processes to ensure the U.S. pharmaceutical system remains one of the safest and most advanced in the world. As has been done with recent examples to respond to emerging public health and regulatory challenges (e.g., sunscreen absorption, carcinogenicity evaluation, opioid drug interactions and respiratory depression, and safety of COVID-19 therapies) the Division’s laboratory, computational, and clinical research expertise, and infrastructure position it to be able to respond to the next challenges the Agency faces. In addition, DARS continues to help make drug development more efficient, as it has done with advancing new methods for biosimilar and complex generic drug evaluations and adopting new technologies to speed the development of treatments for rare diseases. Performing these mission-critical tasks involves complex science, innovative thinking, and a high-level of dedication.

## Author contributions

KC, RRa, RRo, and DS contributed to the development and design of the manuscript. KC and RRa wrote the first draft of the manuscript. All authors wrote sections of the manuscript, contributed to manuscript revision, read, and approved the submitted version.
