# Supplementary material for: New science, drug regulation, and emergent public health issues: The work of FDA’s division of applied regulatory science
Source: Front Med (Lausanne). 2023 Jan 19;9:1109541. doi: 10.3389/fmed.2022.1109541 (PMC9893027; doi:10.3389/fmed.2022.1109541)
Supplement: Supplementary file 1 [file Table_1.docx]

**Appendix 1: DARS Expert Capabilities**

***Omics***

The DARS omics group is a multi-faceted group with expertise in genomics, transcriptomics, proteomics, **molecular biology**, biochemistry, and bioinformatics providing a range of technical capabilities for regulatory research evaluating drug response and toxicity. The group is equipped to conduct quantitative polymerase chain reaction profiling and a wide range of third-generation and next-generation sequencing (NGS). DARS can then apply suitable bioinformatics tools and information technology to process, analyze and interpret omics data.

***Bioanalytical Laboratory***

Bioanalysis is a primary component of DARS mission in support of drug metabolism, pharmacokinetic, and toxicology studies. The bioanalytical group is equipped with various high-end mass spectrometers coupled with ultra-performance liquid chromatographic systems. Capabilities include quantification of small molecule drugs, therapeutic proteins, impurities, and metabolites along with method development and validation for their routine quantitative analysis. When appropriate, robotic sample handling systems allow the use of high-throughput analyses with a variety of biological matrices including plasma, serum, urine, tissue homogenates and advanced sample collection techniques such as dried blood spot. The bioanalytical laboratory performs liquid chromatography tandem mass spectrometry (LC-MS/MS), high-performance liquid chromatography-ultraviolet spectroscopy (HPLC-UV), and enzyme-linked immunosorbent assay (ELISA) analysis and supports a variety of studies including clinical and nonclinical pharmacokinetic-pharmacodynamic modeling, bioequivalence, drug metabolism and transporters, *in-vivo*/*in-vitro* correlations and drug-drug interaction studies.

***Immunology Lab***

DARS immunology research focuses on immunotoxicology, immunopharmacology and immunogenicity of biological products, as well as assay/model development and validation. These areas are addressed using *in vitro* techniques with a range of immunological tools including flow cytometry, enzyme-linked immunosorbent spot (ELISpot), multiplex ELISA platforms and multiplex gene expression analysis. In addition, advanced animal models are used to better understand the immunotoxicology risk of drug products.

***Clinical Informatics and Medical Toxicology***

DARS leverages multiple informatics methodologies, including machine learning and natural language processing methods, to evaluate the pharmacology and biologic plausibility of safety issues for new and marketed drug products. DARS creates and evaluates computational tools to predict the molecular targets a drug may bind to and safety issues that may arise from these interactions. Additionally, natural language processing expands FDA’s available information sources (such as social media) and helps the Agency better leverage and review existing sources of information (such as drug labeling). DARS medical toxicology expertise contributes to ongoing DARS-led clinical trials as well as safety assessments and communications related to off-label drug use.

***Quantitative Systems Pharmacology (QSPs)***

Quantitative Systems Pharmacology (QSPs) modeling is a computational model that characterizes biological and pharmacological systems. DARS is evaluating potential roles for QSP modeling to inform regulatory decision making. Additionally, DARS created a database (1) of QSP submissions to better understand current QSP modeling practices and identify opportunities for QSP in drug development.

***Clinical Trials***

Over the past 5 years, DARS has greatly expanded its capability to run prospective clinical trials to address critical regulatory, drug development and public health questions. The integrated clinical research conducted by DARS covers clinical pharmacology, experimental medicine and postmarket analyses. The capacity to run prospective clinical trials allows for the evaluation of clinical trial methodologies to facilitate new drug development and assess the safety of marketed drugs.

***In Vitro Microphysiological and Cellular Systems***

The DARS integrated cellular systems laboratory is evaluating the use of complex in vitro models (CIVMs) to assess various pharmacokinetic parameters and safety of new and generic drugs. The lab is equipped to use microphysiological systems (MPS) as in vitro models to evaluate drug toxicity, metabolism, and permeability. Currently, DARS is collaborating with academic and industry experts to evaluate the following CIVMs 3D engineered heart tissues, cardiac microphysiological system, liver-on-a-chip (2), gut-on-a-chip and lung-on-a-chip models. In parallel, DARS is assessing the reproducibility of human induced pluripotent stem cells to predict drug safety and efficacy (3).

***Clinical Pharmacology***

The DARS clinical pharmacology laboratory develops *in vitro* assays of drug metabolism or drug transport to predict the need for clinical drug interaction studies and to guide product labeling within FDA’s regulatory guidance. This includes assays for phase I and II drug metabolism for substrates as well as inhibitors or inducers of important enzymes and transporters (4), uptake or bidirectional efflux drug transport assays for evaluating new drugs as substrates or inhibitors of membrane transporters (5), and changes in drug exposure due to an interaction may lead to toxicity or lack of efficacy.

***Safety Pharmacology-Electrophysiology***

The DARS electrophysiology laboratory uses the whole cell patch clamp approach to characterize the effects of drugs on multiple cardiac ion channels. In parallel, the group provides expert consultation to regulatory colleagues regarding the efficacy and safety of investigational products that act on ion channels or alter electrical behaviors of excitable cells.

***Computational Pharmacology and Biology***

DARS develops mechanistic models integrating disease progression, human biology, and quantitative systems pharmacology to evaluate and predict the efficacy and safety of drugs. On behalf of FDA, DARS is collaborating with industry, academia, and other regulatory agencies in developing and implementing international regulatory guidelines regarding the regulatory use of risk prediction models for drug-induced abnormal heart rhythms.

***Computational Toxicology/Pharmacology: (Quantitative) Structure-Activity Relationship [(Q)SAR] Models***

(Quantitative) structure-activity relationship, or (Q)SAR, models describe the association between chemical structural features and biological activity under the assumption that structurally-similar compounds exhibit similar biological activities (6-9). These are particularly useful when limited or no empirical data are available and an assessment of toxicological/pharmacological potential for endpoints is needed. Models and databases developed through DARS are used by the Computational Toxicology Consultation Service to inform and support regulatory decisions for drug products. Additionally, the models are made available externally through commercial (Q)SAR software vendors with whom CDER has formal Research Collaboration Agreements.

**References:**

1. Bai JPF, Earp JC, Florian J, Madabushi R, Strauss DG, Wang Y, et al. Quantitative systems pharmacology: Landscape analysis of regulatory submissions to the US Food and Drug Administration. CPT Pharmacometrics Syst Pharmacol. 2021;10(12):1479-84.
2. Rubiano A, Indapurkar A, Yokosawa R, Miedzik A, Rosenzweig B, Arefin A, et al. Characterizing the reproducibility in using a liver microphysiological system for assaying drug toxicity, metabolism, and accumulation. Clin Transl Sci. 2021;14(3):1049-61.
3. Ribeiro AJS, Guth BD, Engwall M, Eldridge S, Foley CM, Guo L, et al. Considerations for an In Vitro, Cell-Based Testing Platform for Detection of Drug-Induced Inotropic Effects in Early Drug Development. Part 2: Designing and Fabricating Microsystems for Assaying Cardiac Contractility With Physiological Relevance Using Human iPSC-Cardiomyocytes. Front Pharmacol. 2019;10:934.
4. Qosa H, Avaritt BR, Hartman NR, Volpe DA. In vitro UGT1A1 inhibition by tyrosine kinase inhibitors and association with drug-induced hyperbilirubinemia. Cancer Chemother Pharmacol. 2018;82(5):795-802.
5. Volpe DA, Hamed SS, Zhang LK. Use of different parameters and equations for calculation of IC(5)(0) values in efflux assays: potential sources of variability in IC(5)(0) determination. AAPS J. 2014;16(1):172-80.
6. Benigni R, Bossa C. Mechanisms of chemical carcinogenicity and mutagenicity: a review with implications for predictive toxicology. Chem Rev. 2011;111(4):2507-36.
7. Enoch SJ, Cronin MT. A review of the electrophilic reaction chemistry involved in covalent DNA binding. Crit Rev Toxicol. 2010;40(8):728-48.
8. Kazius J, McGuire R, Bursi R. Derivation and validation of toxicophores for mutagenicity prediction. J Med Chem. 2005;48(1):312-20.
9. Mortelmans K, Zeiger E. The Ames Salmonella/microsome mutagenicity assay. Mutat Res. 2000;455(1-2):29-60.
