# Supplementary material for: New science, drug regulation, and emergent public health issues: The work of FDA’s division of applied regulatory science
Source: Front Med (Lausanne). 2023 Jan 19;9:1109541. doi: 10.3389/fmed.2022.1109541 (PMC9893027; doi:10.3389/fmed.2022.1109541)
Supplement: Supplementary file 2 [file Table_2.docx]

**Appendix 2: Current DARS Collaborators***

Abreos Biosciences

AnaBios

Beth Israel Deaconess Medical Center

Chemotargets, SL

CN Bio Innovations, Limited

Dana Solutions, LLC

Emulate, Inc.

John Hopkins University Center of Excellence in Regulatory Science (CERSI)

Harvard Medical School

International Life Sciences Institute (ILSI) Health and Environmental Sciences Institute (HESI)

Leadscope, Inc.

Leiden University Medical Center, the Netherlands

Lhasa Limited

Molecular Health, Inc

National Cancer Institute (NCI), Division of Cancer Treatment and Diagnosis (DCTD), Developmental Therapeutics Clinic (DTC)

National Institutes of Health (NIH), National Center for Advancing Translational Sciences (NCATS)

MultiCASE, Inc.

SomaLogic, Inc.

Spaulding Clinical

UCSF/Stanford Center of Excellence in Regulatory Science (CERSI)

University of Florida, College of Pharmacy

University of Louisville

University of Nottingham and ROCHE

University of Wisconsin, School of Medicine and Public Health

VivaQuant

*List is current as of Oct 22, 2022.
